# Oral Supplementation of Melatonin Protects against Fibromyalgia-Related Skeletal Muscle Alterations in Reserpine-Induced Myalgia Rats

**DOI:** 10.3390/ijms18071389

**Published:** 2017-06-29

**Authors:** Gaia Favero, Valentina Trapletti, Francesca Bonomini, Alessandra Stacchiotti, Antonio Lavazza, Luigi Fabrizio Rodella, Rita Rezzani

**Affiliations:** 1 Anatomy and Physiopathology Division, Department of Clinical and Experimental Sciences, University of Brescia, Viale Europa 11, 25123 Brescia, Italy; gaia.favero@unibs.it (G.F.); segreteria.anatomia@unibs.it (V.T.); francesca.bonomini@unibs.it (F.B.); alessandra.stacchiotti@unibs.it (A.S.); luigi.rodella@unibs.it (L.F.R.); 2 Interdipartimental University Center of Research “Adaption and Regeneration of Tissues and Organs—(ARTO)”, University of Brescia, 25123 Brescia, Italy; 3 OIE Reference Laboratory for RHD, Istituto Zooprofilattico Sperimentale della Lombardia e Emilia Romagna, 25124 Brescia, Italy; antonio.lavazza@izsler.it

**Keywords:** fibromyalgia, inflammation, melatonin, oxidative stress, skeletal muscle

## Abstract

Fibromyalgia is a chronic syndrome characterized by widespread musculoskeletal pain and an extensive array of other symptoms including disordered sleep, fatigue, depression and anxiety. Important factors involved in the pathogenic process of fibromyalgia are inflammation and oxidative stress, suggesting that ant-inflammatory and/or antioxidant supplementation might be effective in the management and modulation of this syndrome. Recent evidence suggests that melatonin may be suitable for this purpose due to its well known ant-inflammatory, antioxidant and analgesic effects. Thus, in the current study, the effects of the oral supplementation of melatonin against fibromyalgia-related skeletal muscle alterations were evaluated. In detail, 90 Sprague Dawley rats were randomly treated with reserpine, to reproduce the pathogenic process of fibromyalgia and thereafter they received melatonin. The animals treated with reserpine showed moderate alterations at hind limb skeletal muscles level and had difficulty in moving, together with significant morphological and ultrastructural alterations and expression of inflammatory and oxidative stress markers in the gastrocnemius muscle. Interestingly, melatonin, dose and/or time dependently, reduced the difficulties in spontaneous motor activity and the musculoskeletal morphostructural, inflammatory, and oxidative stress alterations. This study suggests that melatonin in vivo may be an effective tool in the management of fibromyalgia-related musculoskeletal morphofunctional damage.

## 1. Introduction

Fibromyalgia (FM) is a multifactorial chronic syndrome [1,2] that occurs in up to 6% of the population [3,4]; it is one of the most common pathological conditions seen in primary health care [2,5]. FM is a musculoskeletal condition characterized by chronic pain and tenderness (generalized allodynia/hyperalgesia) accompanied by other somatic and psychological symptoms including fatigue, sleep disturbances, irritable bowel, restless leg, tension or migraine headaches, dysmenorrhea and cognitive difficulties including memory problems, concentration difficulties and psychological and emotional distress [6,7].

Despite significant developments in understanding its pathophysiology, the etiology of FM is still unknown [8]. Liptan [9] underlined the role of a generalized inflammation of the muscle fascia leading to widespread pain and central sensitization and proposed that the fascial dysfunction in FM could be caused by inadequate deep sleep and insufficient growth hormone release. Furthermore, a possible peripheral pathogenic factor involved in FM etiopathogenesis is an imbalance between pro- and anti-inflammatory cytokines [10,11], as this derangement seems, in turn, to play an important role in induction and maintenance of chronic pain [8], fatigue [12] and muscle stiffness [13]. Sprott and colleagues [14] demonstrated that patients with FM are characterized by abnormalities in muscle tissue that include increased DNA fragmentation, impaired expression of constitutive enzymes and changes in the number and size of mitochondria. Regarding this hypothesis, cyclooxygenase-1 (COX-1) seems to be a key enzyme. COX-1 is a constitutive enzymatic isoform involved, at skeletal muscle level, in the regulation of microcirculation, basal turnover and in various stages of myogenesis [15] and so it may also be involved in fibromyalgic pathogenesis. Furthermore, the decrease in mitochondrial mass and the rise in production of mitochondria derived radical oxygen species (ROS) have recently been proposed as relevant events in the fibromyalgia-related alterations [16]. Many studies have noted a correlation between the increase of ROS and the reduction of endogenous antioxidant defenses, including superoxide dismutase (SOD), catalase (CAT) and glutathione, is strictly linked with the symptoms of FM [17]. Recently, nucleotide oligomerization domain like receptor 3 (NLRP3) inflammasomes was found to be related to mitochondrial oxidative stress. ROS production is induced by many NLRP3 inflammasome stimulators and, at the same time, elevated ROS are essential for inflammasome activation [18]. Interestingly, in fibromyalgia patients, after ROS-mediated activation, NLRP3 promotes the production of pro-inflammatory cytokines [19].

The association of FM with inflammation and oxidative stress suggest that ant-inflammatory and/or antioxidant therapy might be important in FM management and modulation. The degree of improvement achieved by many drugs prescribed for FM is modest at best; in fact, 40–60% of patients do not respond to drug therapy [20] and most fibromyalgia patients are sensitive to the side effects of these medications [21]. Consequently, efforts to identify and promote new therapeutic strategies for fibromyalgia patients are still under consideration.

Recent evidence suggests that melatonin may be suitable and useful because of its multitasking properties as shown in several clinical studies [22,23,24]. Melatonin is a small, highly conserved indoleamine with important chronobiological features [25]. In addition to its chronobiological role, additional beneficial effects of melatonin have been reported including antioxidant, ant-inflammatory, antidepressant, sedative and analgesic activities [26,27,28,29,30]. It is a pleiotropic agent that has been proven safe and remarkably well tolerated in human and animal models over a wide range of doses [31].

Data supporting the claim that melatonin may have efficacy as a FM treatment are slight. Herein, we used a validated fibromyalgia animal model, the reserpine-induced myalgia rat (RIM), to obtain data related to this issue. We initially investigated motor activity, morphological, ultrastructural and oxidative stress and inflammatory changes at skeletal muscle level to document the basis of fibromyalgia etiopathogenesis and we studied in detail the involvement of NLRP3 inflammosome in the model of this disease. We also evaluated the effects of melatonin and its mechanism(s) of action to better understand the pathogenesis of this disorder.

The results provide evidence that melatonin, dose and duration of treatment dependently, reduced significantly the difficulties in spontaneous motor activity, alterations in musculoskeletal cytoarchitecture and induction of inflammatory and oxidative stress processes. Regarding the effect of melatonin on the inflammosome NLRP3, we report that its expression is significantly attenuated. Thus, we suggest that melatonin may be useful for minimizing the pathological processes related to FM disease.

## 2. Results

### 2.1. Body Weight Time-Course

All animals survived the treatments. During the interval of reserpine administration (from its first administration until the first day post-reserpine injection), the animals exhibited a significant reduction in food intake (analysis of variance: ANOVA, *p* ≤ 0.05); this adverse event was closely link to reserpine treatment and its severity decreased progressively after the last dose with food intake returning to control levels. There were no significant changes in food consumption among all the experimental groups following reserpine treatment.

A progressive increase in body weight was observed in animals of all experiment groups during the treatment period. The RIM animals, however, showed a significantly slower rate of body weight gain during the period of reserpine treatment, an effect directly associated with the significant reduction of food intake in these animals. This lower rate of weight gain progressively diminished up to one month after treatment with reserpine. The melatonin-treated RIM rats, without differences between the doses of melatonin administration, did not suffer the slower body weight gain observed in RIM rats. Moreover, the animals treated only with 0.5% glacial acetic acid (vehicle for reserpine) or 1% ethanol dissolved in drinking water for up to 60 days (vehicles for melatonin) showed no significant differences in food intake and body weight gain compared to untreated controls.

### 2.2. Voluntary Motor Activity

**Figure 1:**
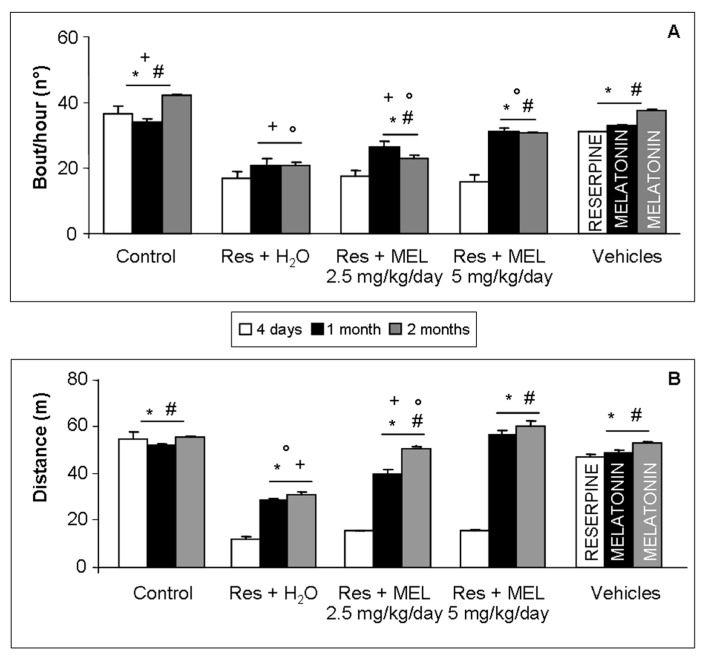
Voluntary locomotor activity. The graphs summarize: the number of spontaneous bouts (**A**); and the distance travelled expressed in meters (**B**) of all experimental groups, evaluated during 1 h of spontaneous locomotor activity. ANOVA, two-way analysis of variance; * *p* ≤ 0.05 vs. Reserpine four days; # *p* ≤ 0.05 vs. Reserpine plus tap water; + *p* ≤ 0.05 vs. Reserpine plus melatonin 5 mg/kg/day for 2 months and ° *p* ≤ 0.05 vs. Controls. H_2_O: tap water; MEL: melatonin; Res: reserpine.

We confirmed that treatment with reserpine duplicated some of the FM symptoms since the motor activity of these rats was significantly reduced compared with controls (ANOVA, *p* ≤ 0.05). Furthermore, we observed that treatment with melatonin, at both doses tested (2.5 mg/kg and 5 mg/kg) and at 30 and 60 days after reserpine administration, led to an improvement in number of spontaneous accesses to the wheel and in running distance traveled, compared RIM animals not given melatonin. In detail, the improvement is significantly higher after the melatonin treatment at the dose of 5 mg/kg and duration two months (ANOVA, *p* ≤ 0.05). It is also important to underline that rats treated with reserpine and then not given melatonin, but only tap water for 30 or 60 days, also exhibited a progressive, but not significant, improvement in bout/hours of voluntary running activity, as observed in rats given melatonin continually. The experimental animals treated only with 0.5% glacial acetic acid (vehicle for reserpine) and with 1% ethanol dissolved in drinking water (vehicle for melatonin) showed no significant differences compared control rats (Figure 1).

For the reported analyses (food intake, body weight and motor activity), the experimental animals treated only with melatonin or reserpine vehicles showed no significant difference compared with age-related untreated controls. For this reason, these experimental groups will be considered in the subsequent analyses as a single group defined generically as “control”. In addition, in the previous reported evaluations, melatonin had no beneficial effects when administered with reserpine (four days); thus, for the analyses of muscle atrophy, inflammatory and oxidative stress markers the experimental groups treated with melatonin for three days were not reported.

### 2.3. Morphological Evaluations on Gastrocnemius

**Figure 2:**
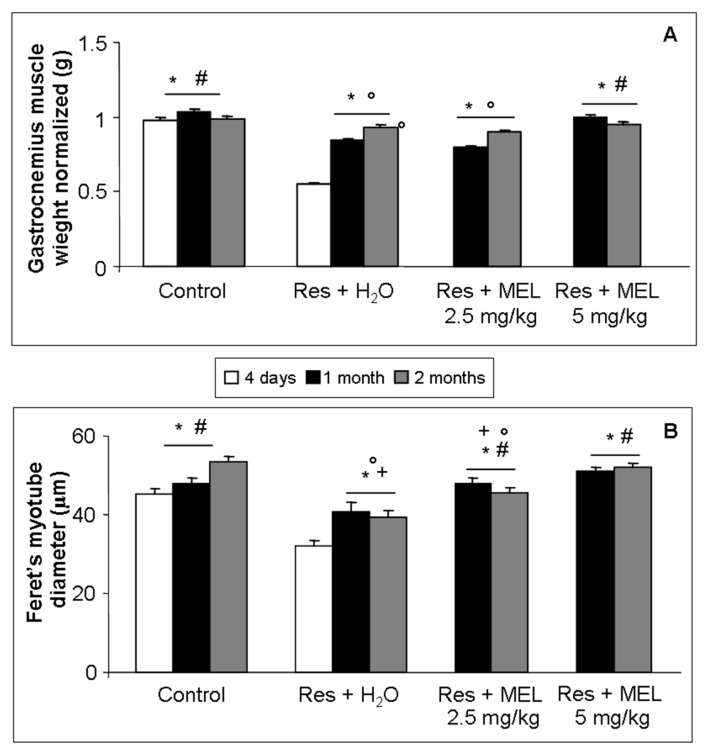
Skeletal muscle atrophy. The graphs summarize: the gastrocnemius muscle weight, normalized to body weight, expressed in grams (**A**); and the Feret’s myotube diameter of gastrocnemius skeletal muscle, expressed in µm (**B**). ANOVA, two-way analysis of variance; * *p* ≤ 0.05 vs. Reserpine four days; # *p* ≤ 0.05 vs. Reserpine plus tap water; + *p* ≤ 0.05 vs. Reserpine plus melatonin 5 mg/kg/day for 2 months and ° *p* ≤ 0.05 vs. Controls. H_2_O: tap water; MEL: melatonin; Res: reserpine.

Treatment with reserpine caused a significant gastrocnemius weight reduction compared with age-related control groups (ANOVA, *p* ≤ 0.05), as expected from RIM experimental animals. Furthermore, treatment with melatonin, at both doses and duration of treatments, caused a significant increment in gastrocnemius weight compared with the RIM group (ANOVA, *p* ≤ 0.05); this improvement is significantly higher at the melatonin dose of 5 mg/kg after both treatment for one month and two months (ANOVA, *p* ≤ 0.05), so it appeared to be mainly dose-dependent. Even rats treated with reserpine and then with only tap water showed some recovery from muscle atrophy, but not significant in respect to melatonin treatment at both doses and duration of treatments (Figure 2A). At the light microscopic level another skeletal muscle atrophy marker, the myotube diameter, was examined. This analyses showed the same trend observed for gastrocnemius weight, briefly, the Feret’s diameter of gastrocnemius myotubes decrease significantly in the experimental group treated with reserpine compared with controls (ANOVA, *p* ≤ 0.05); in contrast, treatment with melatonin had significant beneficial effects, especially when administered at the dose of 5 mg/kg for two months, with respect to RIM experimental group and also with respect to experimental groups treated with reserpine and then only with tap water (Figure 2B).

### 2.4. Ultrastructural Evaluation of Gastrocnemius Muscle

**Figure 3:**
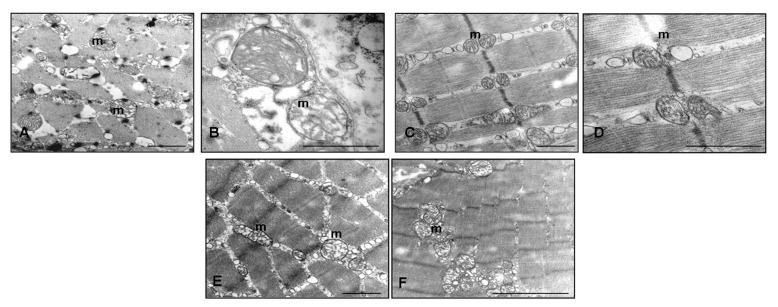
Skeletal muscle ultrastructure. Transmission electron microscopy photomicrographs of: reserpine-induced myalgia rats (**A**,**B**); rats treated with reserpine and then with only tap water for two months (**C**,**D**); and rats treated with reserpine and then with melatonin at the dose of 5 mg/kg/day for two months (**E**,**F**). Scale bar: 1 μm. (m) identifies the mitochondria.

The ultrastructural evaluation of RIM gastrocnemius muscles showed different sarcomeric lengths, heterogeneous and altered interfibrillar and subsarcolemmal mitochondria, and dilated and deformed sarcoplasmic reticulum that appeared associated to mitochondria (Figure 3A,B). Control muscle cells showed normal nuclei with active nucleoli, regular mitochondria distribution and sarcoplasmic reticulum (data not shown). Rats treated with reserpine and then with only tap water for either one or two months of treatment had a weak, but not significant, recovery of the gastrocnemius ultrastructure (Figure 3B,C). However, under melatonin, at both doses and duration of treatments, we observed regular satellite cell nucleus and sarcomeric structure, enhanced mitochondria development and significant reduction of abnormal cristae of both interfibrillar and subsarcollemal mitochondria. In detail, the ultrastructural recovery is significant and evident after melatonin treatment at the dose of 5 mg/kg and two months of duration (ANOVA, *p* ≤ 0.05). Figure 3D,E shows the gastrocnemius muscle of the RIM treated with melatonin at the dose of 5 mg/kg/day for two months, illustrating the main melatonin beneficial effects at ultrastructural level.

### 2.5. TSH Level Assessment

**Figure 4:**
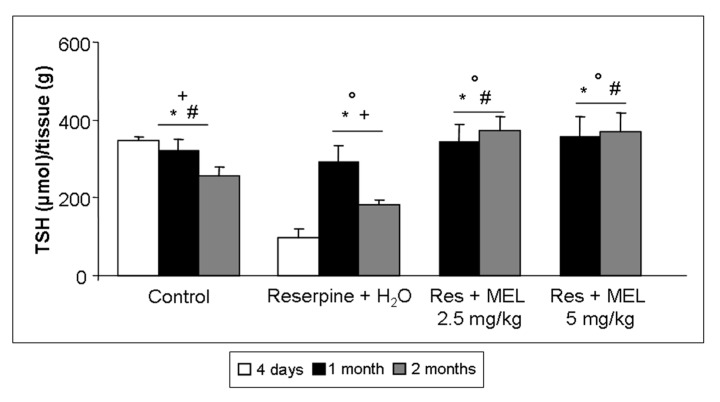
Total thiols. The graph summarizes the gastrocnemius total thiol levels of all experimental groups, expressed as µmol/grams. ANOVA, two-way analysis of variance; * *p* ≤ 0.05 vs. Reserpine four days; # *p* ≤ 0.05 vs. Reserpine plus tap water; + *p* ≤ 0.05 vs. Reserpine plus melatonin 5 mg/kg/day for 2 months and ° *p* ≤ 0.05 vs. Controls. H_2_O: tap water; MEL: melatonin; Res: reserpine; TSH: total thiol.

RIM experimental animals showed significantly lower gastrocnemius TSH level compared to controls (ANOVA, *p* ≤ 0.05). We observed also that treatment with melatonin, at both doses and duration of treatments (without significant differences among dose or duration of melatonin treatment), increased significantly the levels of TSH in the gastrocnemius muscle compared rats treated only with reserpine (ANOVA, *p* ≤ 0.05). Interestingly, the TSH levels observed in the experimental group treated with reserpine and melatonin, at both doses and duration of treatment, were significantly higher than the values observed in age-related controls (ANOVA, *p* ≤ 0.05). Probably, this increment of TSH level depends on the activation of compensatory mechanism(s) against FM—induced oxidative stress. The increment of TSH level in skeletal muscle was observed also in the experimental groups treated with reserpine, but significantly lower relative to melatonin treatments (ANOVA, *p* ≤ 0.05). Control rats after two months showed significantly lower levels of TSH than the control group at one month (ANOVA, *p* ≤ 0.05), which may be related to physiological aging and oxidative stress. In Figure 4, the TSH gastrocnemius levels of all experimental groups are summarized.

### 2.6. Inflammatory and Oxidative Stress Markers Analyses

**Figure 5:**
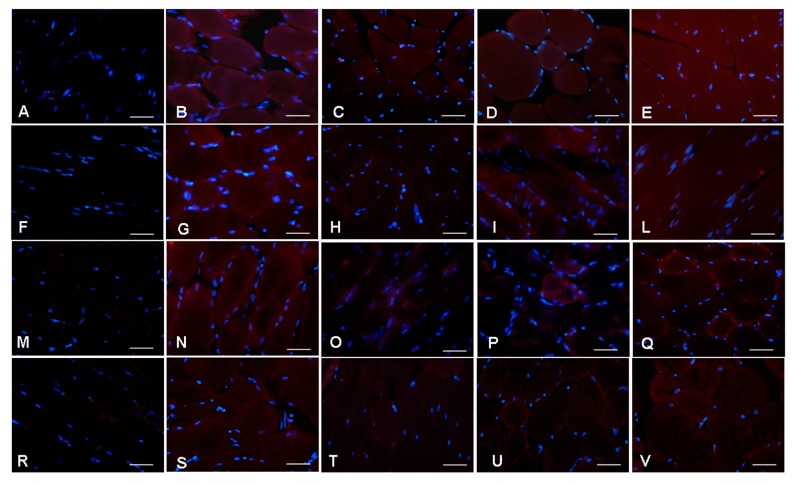
Skeletal muscle oxidative stress and constitutive markers expression. Immunofluorescence photomicrographs of: gastrocnem us skeletal muscle superoxide dismutase 1 (**A**–**E**); catalase (**F**–**L**); cyclooxygenase-1 (**M**–**Q**); and sirtuin 3 (**R**–**V**) expression of: reserpine-induced myalgia rats (**A**,**F**,**M**,**R**); controls (**B**,**G**,**N**,**S**); rats treated with reserpine for two months (**C**,**H**,**O**,**T**); rats treated with reserpine and then with melatonin at the dose of 2.5 mg/kg/day for two months (**D**,**I**,**P**,**U**); and rats treated with reserpine and then with melatonin at the dose of 5 mg/kg/day for two months (**E**,**L**,**Q**,**V**). Nuclei were stained with DAPI (blue). Scale Bar: 20 µm. The quantitative measurement of the immunopositivity of both antioxidant enzymes and constitutive molecules of all experimental animals are shown in Figure 6A–D.

At the gastrocnemius level, we observed that the RIM experimental rats had significantly decreased expression of endogenous antioxidant enzymes (SOD1 and CAT) and of constitutive molecules, also involved in inflammatory, oxidative stress, aging and myogenesis processes (COX-1 and SIRT3) (ANOVA, *p* ≤ 0.05). Treatment with melatonin induced a significant increase of SOD1 expression (red staining) (ANOVA, *p* ≤ 0.05) and this significant enhancement was observed also after treatment with reserpine and tap water (ANOVA, *p* ≤ 0.05); however, treatment with 2.5 mg/kg/day of melatonin for one month did not show significant differences compared with age-related animals treated with reserpine and tap water (Figure 5A–E). The analysis of immunopositivity for CAT (red staining) showed a trend similar to that observed for SOD1, even though the melatonin beneficial effects is significantly higher after two months of treatment (ANOVA, *p* ≤ 0.05), but not showed difference due to the duration of treatment. Moreover, the experimental rats treated with reserpine and tap water showed a significantly lower immunopositivity compared with the animals treated with melatonin (ANOVA, *p* ≤ 0.05); only the rats of two months showed a significantly higher immunopositivity than the RIM experimental animals (ANOVA, *p* ≤ 0.05) (Figure 5F–L).

We also analyzed COX-1 (red staining) and observed that in RIM animals it was significantly reduced, while its expression after treatment with melatonin is significantly higher after melatonin treatment at the dose of 5 mg/kg and duration of two months (ANOVA, *p* ≤ 0.05), reaching a level comparable to that in the age-related control animals. It is important to underline also that the melatonin treatment with 2.5 mg/kg/day showed similar COX-1 immunopositivity compared with the experimental groups treated with reserpine (Figure 5M–Q). We analyzed also the constitutive molecule SIRT3 (red staining) and we observed that the trend of its immunopositivity is similar to COX-1 expression, although the control group two months showed significant lower levels compared with the control rats at one month (ANOVA, *p* ≤ 0.05). This decrease may be related to physiological aging process. As for COX-1, the main effect of melatonin treatment is observed at the dose of 5 mg/kg administered for two months and the treatment with melatonin at the dose of 2.5 mg/kg/day showed similar levels of immunopositivity compared with age-related group treated with reserpine (Figure 5R–V). Figure 5A–V shows only the immunofluorescence data of gastrocnemius muscle of the experimental animals treated for two months.

**Figure 7:**
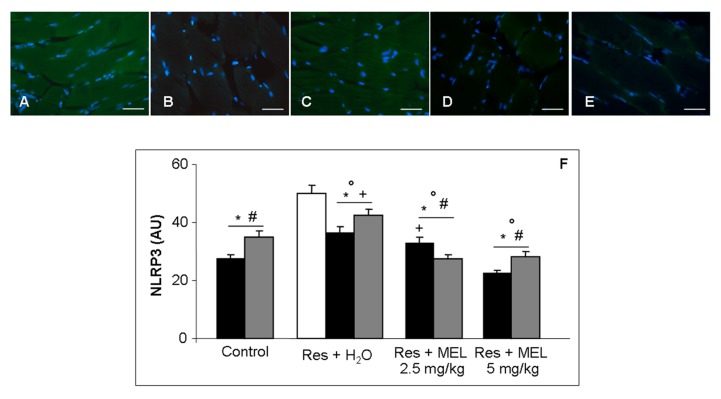
Skeletal muscle inflammatory markers. Immunofluorescence photomicrographs of gastrocnemius muscle inflammosome NLRP3 expression of: reserpine-induced myalgia rats (**A**); controls (**B**); rats treated with reserpine for two months (**C**); rats treated with reserpine plus melatonin at the dose of 2.5 mg/kg/day for two months (**D**); and rats treated with reserpine and then with melatonin at the dose of 5 mg/kg/day for two months (**E**). Nuclei were stained with DAPI (blue). Scale Bar: 20 µm. The graph summarizes the histomorphometrical analyses, expressed in arbitrary units (AU), of inflammosomeNLRP3. ANOVA, two-way analysis of variance; * *p ≤* 0.05 vs. Reserpine four days; # *p* ≤ 0.05 vs. Reserpine plus tap water; + *p* ≤ 0.05 vs. Reserpine plus melatonin 5 mg/kg/day for 2 months and ° *p* ≤ 0.05 vs. Controls. H_2_O: tap water; MEL: melatonin; Res: reserpine.

Finally, we estimated that NLRP3 (green staining) is significantly expressed in rats treated with reserpine compared with the controls (ANOVA, *p* ≤ 0.05); moreover, treatment with melatonin at both doses and duration of melatonin treatments induced a significant reduction of the expression of this inflammasome (ANOVA, *p* ≤ 0.05). The experimental rats treated with reserpine and then with tap water showed a significant decrease of the expression of NLRP3 respect RIM (ANOVA, *p* ≤ 0.05); this decrease was significantly less compared with the groups treated with melatonin (ANOVA, *p* ≤ 0.05). Furthermore, we observed that there are no significant differences between the melatonin treatment at 2.5 mg/kg and 5 mg/kg for two months and that the expression of NLRP3 in control animals two months is greater compared with the expression in rats at one month (Figure 7A–F).

## 3. Discussion

Herein, we showed that the reserpine treated animals presented: (1) a significant decrease in number of spontaneous bouts and running distance traveled; (2) skeletal muscle atrophy due to the alteration of the gastrocnemius weight and diameter of myotubes and significant musculoskeletal ultrastructural alterations; (3) significant rise of TSH and, interestingly, also NLRP3 expressions in skeletal muscle levels; and (4) significant reduction of expression of endogenous antioxidant enzymes (SOD1 and CAT) and of constitutive molecules involved in inflammation, oxidative stress and myogenesis processes (COX-1 and SIRT3). Similar results have also been recorded in previous studies [1,32,33] in which reserpine treatment causes a reduction in locomotor activity that could be related to long-lasting muscular mechanical hyperalgesia, tactile allodynia and tenderness, but also to depression characteristic of FM pathogenesis [34]. Moreover, Blasco-Serra and colleagues [35] documented the depressive-like symptoms in RIM group; they observed the rats had significant aversion to eating in a novel environment. In our study, rats treated with reserpine showed a clear decrease in food consumption and consequently in body weight. In addition, the atrophy of muscle fiber described by Bonaterra et al. [32] is consistent with our results. According other authors [19,36], the underlying mechanisms of these alterations could be inflammation and oxidative stress processes characteristic of FM and also in the RIM model. In particular, our data provide information on the possible mechanisms showing an increase in morphological damage caused by ROS and a reduction in endogenous antioxidants, including SOD and CAT, in gastrocnemius. We also evaluated COX-1, a constitutive marker of myogenesis in different muscles including cardiomyocytes [15]. In particular, we showed that COX-1 was decreased in RIM animals, as already reported by De Almeida and colleagues [37]. Therefore, we suggest that COX-1 has an important musculoskeletal protective role. Moreover, we studied SIRT3 expression and we demonstrated it to be highly expressed in RIM rats. Considering its role in redox regulation by deacetylating mitochondrial proteins like acetyl-coenzyme A synthetase 2, glutamate dehydrogenase and SOD [38], we propose that it has important protective effects against oxidative stress also in FM syndrome [39]. Related to this finding, we noted that the levels of SOD1 and SIRT3 of the control rats at two months were lower compared with control group at one month and, in agreement with other authors [40], we considered this reduction to be a result of physiological aging and oxidative stress process.

After obtaining these data and, in particular, considering the morphological and ultrastructural alterations of mitochondria, we analyzed the expression of NLRP3, which is a prominent marker of mitochondrial ROS generation and inflammation processes [19]. Interestingly, NLRP3 is increased in rats treated with reserpine but not in the control animals at two months, due to age-related alterations. It is known that NLRP3 promotes inflammation, oxidative stress and cell death through the activation of inflammatory caspase 1 and caspase 5 [41]. Our findings suggest that NLRP3 is important for progression of fibromyalgic disease as well as in diabetic cardiopathy [42] and renal dysfunction [43].

However, in addition to an adequate fibromyalgic-animal model that is needed for the identification of markers related to pathogenesis, it is also very important to study appropriate treatments and pharmacological therapies against FM. Thus, the present study also evaluated the potential beneficial effects of melatonin administration against the alterations induced in the RIM model. In accordance with several previous studies conducted with fibromyalgic patients [4,24,44,45,46], our results showed that melatonin reduces significantly the damage induce by FM pathogenic process. In fact, we observed that the treatment with melatonin improved the in volountary motor activity compared with the RIM experimental group and with the group treated with reserpine and then with only tap water. Probably, melatonin treatment causes an improvement in rat’s spontaneous running activity because of its antioxidants and ant-inflammatory mechanisms of action, well known melatonin properties [47,48,49,50,51]. Although these beneficial effects are potentially related also to analgesic [30,52,53,54,55] melatonin properties, experimental studies as well as clinical trials in humans have demonstrated that melatonin has an analgesic properties in chronic, acute, inflammatory and neuropathic pain conditions, including fibromyalgia [44,56,57,58]. Importantly, these effects seem MT2 receptor-mediated, as shown by the group of Granados-Soto describing that selective MT2 receptor partial agonists have analgesic properties through modulation of brainstem descending anti-nociceptive pathways [28,59,60]. Furthermore, in our study, we evaluated the effects of melatonin treatment on skeletal muscle cell atrophy induced by reserpine observing that melatonin causes a significant increment in muscle weight and in myotubes diameter compared with RIM experimental group and the animals treated with reserpine and then only with tap water. In addition previous studies reported that melatonin reduces skeletal muscle atrophy and oxidative stress [49,61,62]. In order to confirm the antioxidant, ant-inflammatory and protective roles of melatonin, we evaluated the skeletal muscle expression of endogenous antioxidant and of constitutive molecules and we found that melatonin modulates muscoloskletal ultrastructure, increases expression of SOD1, CAT, COX-1 and SIRT3 and decreaes NLRP3 compared with RIM experimental group and also with the groups treated reserpine and then only with tap water.

**Figure 8:**
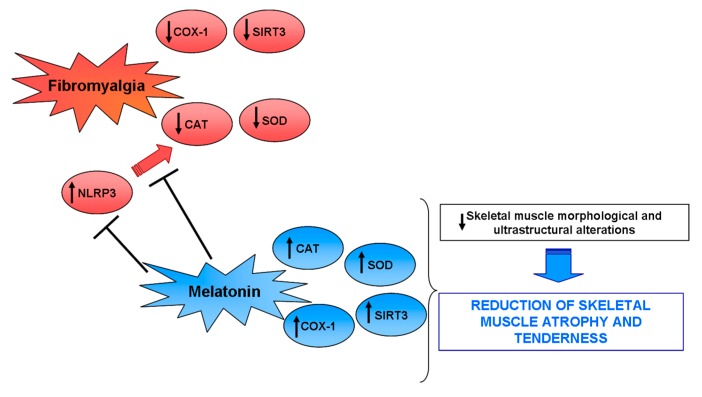
Potential melatonin mechanism(s) of action. A proposed mechanism by which melatonin protects the gastrocnemius skeletal muscle against fibromyalgic alterations through the block of NLRP3 activation. Up arrow indicates an increase in the expression, whereas the down arrow a decrease.

In summary, we suggest that melatonin thought its important inhibiting effect against NLRP3 activation together with its known antioxidant, ant-inflammatory and analgesic properties may block the fibromyalgic pathological processes (Figure 8).

However, further studies on this topic are mandatory to better assess the potential melatonin mechanism(s) of action.

## 4. Materials and Methods

### 4.1. Animal Treatment

90 Sprague Dawley male rats (4–5 weeks old) were housed in standard cages located in a temperature-controlled animal facility (+20 °C) with a 12-h/12-h light-dark cycle for a period up to 3 months. Before the beginning of the treatment with reserpine or melatonin, rats were left housed in the animal facility for at least 1 week. The RIM animal model is based on the hypothesis that dysfunction of biogenic amine-mediated control in the central nervous system (CNS) leads to a disease condition mimicking FM [34,63,64]. In fact, reserpine is an indole alkaloid that depletes catecholamine and so blocks irreversibly the vesicular monoamine transport, causing a marked reduction in the amount of dopamine, norepinephrine and serotonin in various brain regions inducing, in turn, muscle hyperalgesia and tactile allodynia which persist for one week or longer and increases immobility time, an indicator of pain and depression [65]. In the present study, reserpine was injected subcutaneously into the back of rats once a day for 3 consecutive days at a final dose of 1 mg/kg body weight; it was dissolved in 0.5% glacial acetic acid [63,64,66]. Since reserpine treatment may cause a significant decrease in food consumption, during the treatment food pellets was placed directly in the cages, so the RIM experimental animals could feed with less difficulty.

The treatment with melatonin (Melapure™ kindly provided by Flamma S.p.A., Chignolo d’Isola, Italy), in combination or not with reserpine, was administered in two different dosages and three different duration of treatments: melatonin was administrated orally for three days (simultaneously to the treatment of reserpine), or for one month or for two months at a final dose of 2.5 mg/kg body weight for day [67] or at a final dose of 5 mg/kg body weight for day [68]. Powdered pure melatonin was given after being dissolved in 1% of ethanol and then in drinking tap water. In addition to the groups identified above, in the current study were also assessed rats without any treatment (control), rats treated with reserpine and then with only tap water for one month or two months, rats treated for three days with subcutaneous injection of 0.5% glacial acetic acid (vehicle of treatment with reserpine) and rats treated orally with 1% ethanol dissolved in drinking water for one month or two months (vehicles of treatment with melatonin).

The animals of all experimental groups were monitored for weight gain, food consumption and spontaneous motor activity. At the end of treatments period, the animals were killed by decapitation and both gastrocnemius muscles were carefully removed, weight (normalized to body weight) and processed for morphometric, ultrastructural, sulfydryl group (SH) level and immunofluorescence analyses.

### 4.2. Assessment of Volountary Locomotor Activity

The present study used a non-reflexive measures of locomotor activity that require voluntary decision and integrations of multiple CNS centers. The running wheel is of particular interest as a motor test in experimental animals because the locomotion is not forced and potentially reflects whether the activity is painful [69,70]. Voluntary locomotor activity was assessed in polycarbonate cages with free access to stainless steel activity wheels (Bioseb, In Vivo Research Instruments, Vitrolles, France). The wheel (diameter 23 cm; width 5 cm) could be turned in both direction and it is connected to an analyzer that automatically recorded the running activity. The animals had food and water available *ad libitum*. In this study we evaluated the distance travelled (expressed in meters) and the number of spontaneous accesses to the wheel during 1 h of evaluation session for each animal. No experimenters were present in the room during the recording period. Rats were habituated in an individual activity cage for three sessions over at least three days [33]. A baseline measurement was recorded one day after the last habituation. The rats that refused to run in the wheel during the baseline measurement were discarded from further evaluation (representing 2–4% of the animals tested).

All the protocols were approved by the Animal Care and Use Committee (OPBA) of the University of Brescia (Brescia, Italy) and by the Italian Ministry of Health (558/2015-PR-22/06/2015) and comply the commonly-accepted “2Rs” indication.

### 4.3. Total Thiol Evaluation

The total thiol (TSH) levels of gastrocnemius samples were evaluated as previously described by Oliveira and colleagues [71,72]. Briefly, 200 µL of proteic supernatant fraction were precipitated with 200 µL of 4% trichloroacetic acid (*v*/*v*) by centrifugation (1050× *g* for 10 min). Then, the colorimetric analyses were carried out in 1 M phosphate-buffered, pH 7.4. A curve using glutathione as standard was constructed to calculate the total TSH of each sample.

### 4.4. Morphometrical Analysis

Gastrocnemius samples were fixed in 4% buffered paraformaldehyde for 24 h, dehydrated in progressive ethanol solutions, xylene and embedded in paraffin wax, following the standard procedures. Subsequently, 7 mm-thick paraffin sections were cut with a microtome and so the serial paraffin sections were dewaxed in xylene, rehydrated through decreasing scale of ethanol and stained with haematoxylin-eosin, following standard protocol. Then the sections were observed with an optical light microscope (Olympus, Deutschland, Germany) at the final magnification of 200×. Digital images of gastrocnemius were captured and the Feret’s diameter (expressed in µm) of 50 myotubes for each animal muscle was determined using an image analyser (Image Pro Premier 9.1, Media Cybernetics, Rockville, MD, USA) by two observers blinded to the treatment, whose evaluation was assumed to be correct if the values were not significantly different. In case of dispute concerning interpretation, the case was reconsidered to reach an agreement [73,74].

### 4.5. Ultrastructural Transmission Electron Microscopy Evaluation

A piece of gastrocnemius muscle of each rat was treated for ultrastructural analysis [74]. Briefly skeletal muscle tissue was fixed by immersion in 2.5% glutaraldehyde in cacodilate buffer 0.1 M (pH 7.4) for 3 h at +4 °C and postfixed in 2% osmium tetroxide in cacodilate buffer for 1 h at +4 °C. Dehydration process was performed in increasing ethanol concentrations and propylene oxide, then the samples were embedded in Araldite-Epon resin. Semithin sections (1 µm-thick) were collected at an UltraCut E ultramicrotome stained by toluidine blue and observed at a light microscope (Olympus, Deutschland, Germany) to assess the morphological structure of the skeletal muscle. Subsequently, from representative blocks, 70–80 nm-thick ultrathin sections were obtained using a diamond knife, collected on formvar coated copper grids, double stained with uranyl acetate and lead citrate and observed under a transmission electron microscopy (Tecnai G2 Spirit) at 80 kV.

### 4.6. Immunofluorescence Evaluations

Serial paraffin sections were dewaxed in xylene, rehydrated through decreasing scale of ethanol and then washed with phosphate buffer solution 1× (PBS 1×). The blocking step was performed by incubating gastrocnemius sections with specific serum (diluted 1:50 in PBS 1×) for 1 h in a humid chamber. Subsequently, the sections were incubated 1 h at room temperature and then overnight at 4 °C in a humid chamber with the following primary antibodies: rabbit polyclonal antibody against superoxide dismutase 1 (SOD1, diluted 1:300, Santa Cruz Biotechnology Inc., Dallas, TX, USA), goat polyclonal antibody against catalase (CAT, diluted 1:200, Santa Cruz Biotechnology Inc., Dallas, TX, USA), rabbit polyclonal antibody against sirtuin3 (SIRT3, diluted 1:200, Santa Cruz Biotechnology Inc., Dallas, TX, USA), mouse monoclonal antibody against cyclooxygenase 1 (COX-1, diluted 1:100; Cayman Chemical, Ann Arbor, MI, USA) and rabbit polyclonal antibody against Nod-like receptor protein 3 antibody against (NLRP3, diluted 1:650, Novus Biologicals, Milano, Italy). Sections were then washed in PBS 1× and labelled using specific conjugated secondary antibodies (diluted 1:200 in PBS 1×; Invitrogen, Paisley, UK): goat anti-rabbit Alexa Fluor-488, goat anti-rabbit Alexa Fluor 546, rabbit anti-goat Alexa Fluor 546 and goat anti-mouse Alexa Fluor 546. Finally, the samples were counterstained with 4′,6-diamidino-2-phenylindole (DAPI) for 8 min [75,76]. The samples were mounted with fluorescent medium and observed with a fluorescent microscope (i50 Eclipse, Nikon, Düsseldorf, Germany) at final magnification of 400× [71]. Sections without primary antibody and in the presence of isotype-matched IgG served as negative immunofluorescent controls.

Twenty random fields, each with an area of 0.04 mm^2^, from a total of five sections for each rat’s gastrocnemius were analyzed and the immunostaining for each primary antibody were calculated using an image analyzer (Image Pro Premier 9.1, MediaCybernetics, Rockville, MD, USA). Two blinded investigators, whose evaluation was assumed to be correct if the values were not significantly different, made the evaluation of positive immunostaining. In the case of dispute concerning interpretation, the case was reconsidered to reach an agreement [73,74].

### 4.7. Statistical Analysis

The data obtained in the current study were presented as means ± SD. Statistical analyses were performed using a two-way analysis of variance test corrected by Bonferroni, evaluating both dose and duration of treatments as different variable. Differences among groups were considered statistically significant at *p* ≤ 0.05.

## Figures and Tables

**Figure 6:**
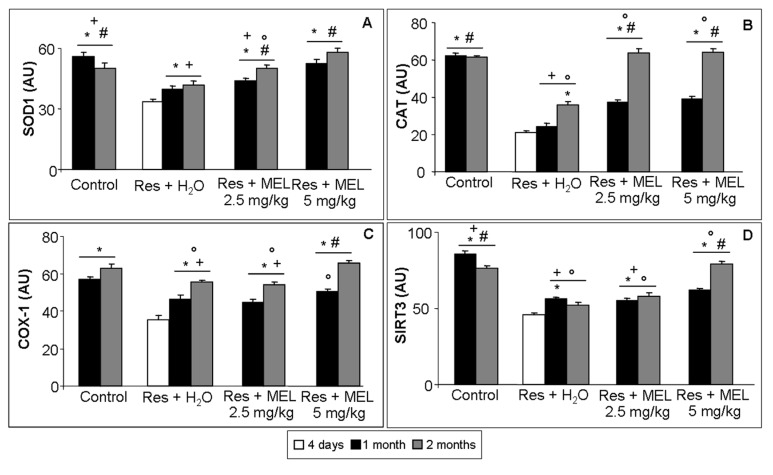
Skeletal muscle oxidative stress and constitutive markers quantitative analyses. The graphs summarize the histomorphometrical analyses expressed in arbitrary units (AU) of: superoxide dismutase 1 (SOD1) (**A**); catalase (CAT) (**B**); cyclooxygenase-1 (COX-1) (**C**); and sirtuin 3 (SIRT3) (**D**) immunopositivity at gastrocnemius skeletal muscle level. ANOVA, two-way analysis of variance; * *p* ≤ 0.05 vs. Reserpine four days; # *p* ≤ 0.05 vs. Reserpine plus tap water; + *p* ≤ 0.05 vs. Reserpine plus melatonin 5 mg/kg/day for 2 months and ° *p* ≤ 0.05 vs. Controls. H_2_O: tap water; MEL: melatonin; Res: reserpine.
